# Inflammatory Biomarkers and Blood Physical Property Transformations Following On-Pump Coronary Artery Bypass Graft Surgery

**DOI:** 10.3390/jpm13101434

**Published:** 2023-09-26

**Authors:** Po-Shun Hsu, Jia-Lin Chen, Shih-Ying Sung, Yi-Ting Tsai, Chih-Yuan Lin, Yi-Fan Wu, Chien-Sung Tsai

**Affiliations:** 1 Division of Cardiovascular Surgery, Department of Surgery, Tri-Service General Hospital, National Defense Medical Center, Taipei 114202, Taiwan; 2 Department of Anesthesia, Tri-Service General Hospital, National Defense Medical Center, Taipei 114202, Taiwan; 3 School of Dentistry, College of Oral Medicine, Taipei Medical University, Taipei 110301, Taiwan; 4 Institute of Physics, Academia Sinica, Taipei 115201, Taiwan

**Keywords:** cardiopulmonary bypass, on-pump coronary artery bypass graft, inflammation, hemorheology

## Abstract

Objective: This study aimed to compare the hemorheological and inflammatory changes before and after coronary artery bypass graft (CABG) surgery, as factors such as hypothermia, hemodilution, transfusion, and other variables affect blood viscosity and inflammation during the procedure. Methods: A total of 25 patients who underwent CABG surgery were enrolled in this study. Whole blood was collected just before the CABG (D_0_), 2 days after surgery (D_2_), and 5 days after surgery (D_5_). The plasma viscosity (PV) and whole blood viscosity (WBV) were measured at shear rates ranging from 0.1 to 1000 s^−1^ using a rheometer, and the mean values were compared. Inflammatory markers were also assessed and analyzed in relation to the hemorheological changes. Results: Compared with the baseline values, the PV significantly increased after 5 days. WBV showed a significant increase on day 2 and after 5 days. The WBV and fibrinogen were significantly correlated on day 2 and day 5 but not before surgery. Inflammatory markers such as CRP, WBC, platelets, and fibrinogen also demonstrated notable changes in relation to the hemorheological alterations. Conclusions: This study highlights the crucial finding that hyperviscosity, characterized by elevated PV and WBV, persists for almost one week after on-pump CABG surgery. Understanding the interplay between inflammation and hemorheological properties during the postoperative period is crucial for optimizing patient care. Future research should focus on exploring the underlying mechanisms and potential therapeutic interventions to mitigate the impact of inflammation on blood viscosity and improve patient outcomes following CABG surgery.

## 1. Introduction

Coronary artery bypass grafting (CABG) is a frequently administered surgical procedure designed to enhance cardiac blood flow by redirecting it through grafts from other vessels around obstructed or narrowed coronary arteries [1]. While outcomes are generally favorable, with 85% of patients reporting significant symptom reduction and decreased mortality risk within a decade post-surgery, CABG is not without its risks [2]. CABG surgery is often considered a high-stakes procedure, with 30 day morbidity and mortality rates as high as 14.0% and 2.0%, respectively [3].

The cardiopulmonary bypass (CPB), commonly referred to as the heart-lung machine, is frequently employed during CABG to momentarily take on the roles of the heart and lungs, facilitating a motionless, bloodless surgical field. However, this procedure is often viewed as high-risk, with potential complications such as stroke, further surgery necessitated by bleeding, myocardial infarction, arrhythmias, and deep sternal wound infection, each with a risk rate of around 1–2% [4,5]. Blood viscosity fluctuates during cardiac surgery with a CPB due to hypothermia and hemodilution [6]. These risks can be influenced by inflammation and changes in blood rheological properties, both of which can be affected by non-endothelial cell surface exposure, triggering the release of certain hormones and inflammatory cytokines [7].

Inflammation plays a critical role in cardiovascular disease (CVD) pathophysiology and post-CABG complications. Tracking inflammatory biomarkers such as C-reactive protein (CRP), interleukins (ILs), and tumor necrosis factor-alpha (TNF-α) in CABG patients could provide valuable insights into a patient’s inflammatory status and potential for postoperative complications [8]. Moreover, the intricate relationship between inflammation and hemorheological changes is increasingly acknowledged as an influential factor in post-surgery recovery and complication risks [9,10]. Recent studies have shown associations between altered hemorheological properties and increased complications in CVD patients, stressing the importance of understanding these connections for clinical decision making and targeted intervention development. For example, a study by Sloop et al. [11] found that increased blood viscosity was linked to a higher risk of myocardial infarction in the acute phase, while Schmidt et al. [12] reported that blood viscosity was associated with carotid artery intima-media thickness, a marker of early atherosclerosis. Exploring these connections can potentially guide clinical decision making and the development of targeted interventions to optimize patient outcomes following CABG.

Currently, while inflammation is acknowledged as a significant contributor to post-CABG complications, the specific interactions between inflammation, blood viscosity, and their impact on patient outcomes are not fully understood. To address this knowledge gap, the present study investigates changes in blood viscosity following CABG surgery with CPB, focusing on the relationship between fibrinogen, an acute-phase reactant, and the whole blood viscosity (WBV). It emphasizes the importance of comprehensively understanding the interplay between inflammatory biomarkers, blood viscosity changes, and CABG complications in patient care optimization and clinical decision making. Ultimately, this research aims to shed light on the associations between inflammatory biomarkers, hemorheological properties, and their influence on postoperative complications, thus contributing to the development of personalized treatment strategies to mitigate complications and improve patient outcomes following CABG surgery.

## 2. Material and Methods

### 2.1. Study Approval and Patient Enrollment

The study protocol was sanctioned by the Institutional Review Board (TSGHIRB No: 1-104-05-032), and written informed consent was obtained from all patients prior to the CABG. This study recruited 25 patients, encompassing 8 women and 17 men, necessitating CPB assistance for on-pump CABG. The demographic details and preoperative characteristics of the participants are catalogued in Table 1.

The average age of the patients was 63.1 ± 3.1 years, the median age was 61 years (range: 25–65 years), and the median body mass index (BMI) was 24.16 ± 0.81 kg/m^2^. Among the cohort, hypertension was observed in 16 patients (64%), hyperlipidemia was observed in 15 patients (60%), and diabetes was observed in 9 patients (36%). Antiplatelet drugs were discontinued 5 days before CABG and subsequently resumed on the day after the operation.

### 2.2. CABG Surgical Procedure and Post-Operative Care

CPB initiation involved a median sternotomy and systemic heparinization in maintaining an appropriate activated clotting time. Arterial perfusion was facilitated via the cannulation of the ascending aorta, and a two-stage cannula enabled venous drainage. Mild hypothermia (32–34 °C) was maintained throughout the procedure. Myocardial protection was secured by administering the Plegisol solution every 20 min through both antegrade and retrograde coronary perfusion. Post bypass completion, ultrafiltration facilitated hemoconcentration to restore hematocrit levels >30% and eliminate intravascular and extravascular water and inflammatory substances. The hematocrit, blood gases, acid-base balance, and plasma electrolytes were evaluated and regulated. Once satisfactory and stable cardiac function was confirmed via transesophageal echocardiography, all catheters and cannulas were removed, and protamine was administered to reverse heparinization. Following adequate hemostasis, the patients were transferred to the cardiac intensive care unit (ICU). Post-surgery fluid management involved blood product transfusion (packed red blood cells, fresh frozen plasma, and platelet concentrate) to maintain hematocrit levels around 30% (D_2_ hematocrit in Table 2) for instances of excessive bleeding. Crystalloid solutions were utilized to maintain optimal hemodynamic stability.

### 2.3. Blood Sampling, Biochemistry, and Hemorheology

The hemorheological properties were evaluated for each patient just before CABG (D_0_), 2 days post-surgery (D_2_), and 5 days post-surgery (D_5_). Blood samples were collected in ethylenediaminetetraacetic acid (EDTA) and trisodium citrate tubes, and the PV and WBV were measured within 2 h using a Physica rheometer (MCR301, Anton-Paar GmbH, Graz, Austria) with a titanium concentric cylinder. All experimental measurements were conducted at 37 °C to remove any discrepancies related to temperature. The new correction method was employed to standardize all WBV values to a hematocrit of 45%, accounting for patient hematocrit level variations [13].

Furthermore, cardiovascular risk factors and laboratory findings influencing blood viscosity were recorded, including hemoglobin, hematocrit, white blood cell count, platelet count, prothrombin time, partial thromboplastin time, international normalized ratio, fibrinogen, and C-reactive protein. This comprehensive data analysis allowed for more accurate comprehension of the factors impacting blood viscosity during the perioperative period and patient recovery post CABG surgery.

## 3. Statistical Analysis

Statistical analysis of the results was performed using the mean values ± standard deviation (mean ± SD). Differences in the biochemical and rheological parameters were statistically analyzed using the Student’s *t* test for pairwise comparisons and one-way analysis of variance (ANOVA) for multiple groups, with post hoc comparisons conducted using Tukey’s HSD, contingent upon the data meeting the assumptions of normality and homogeneity of variance. All tests were two-tailed, with a significance level set at *p* < 0.05. The Pearson correlation coefficient was calculated to assess the relationship between continuous variables to ensure a reliable understanding of the blood property during the perioperative period and recovery of the patients undergoing CABG surgery.

## 4. Results

### 4.1. Grafting Details and Post-Operative Care

The mean graft per patient was 3.81 ± 0.23, and the left internal mammary artery (LIMA) was employed in 23 of the 25 patients. The average cross-clamping time was approximately 55.4 ± 13.1 min, and the CPB duration averaged 124.0 ± 8.3 min (Table 2). During the intensive care unit (ICU) stay, no patient required a blood transfusion or re-exploration due to bleeding. The duration of mechanical ventilation averaged 1.40 ± 0.32 days. One patient experienced lung atelectasis, and two patients exhibited acute kidney injury, which was delineated by an increase in serum creatinine levels of more than 0.3 mg/dL during their ICU stay. The mean ICU duration was 2.75 ± 0.98 days. No significant complications were noted, and all patients were discharged successfully. The mean duration of hospitalization was 10.90 ± 4.18 days. These results confirm the efficacy and safety of the applied surgical technique and post-operative management for CABG patients in this study.

### 4.2. Hemorheological Characteristics

**Figure 1:**
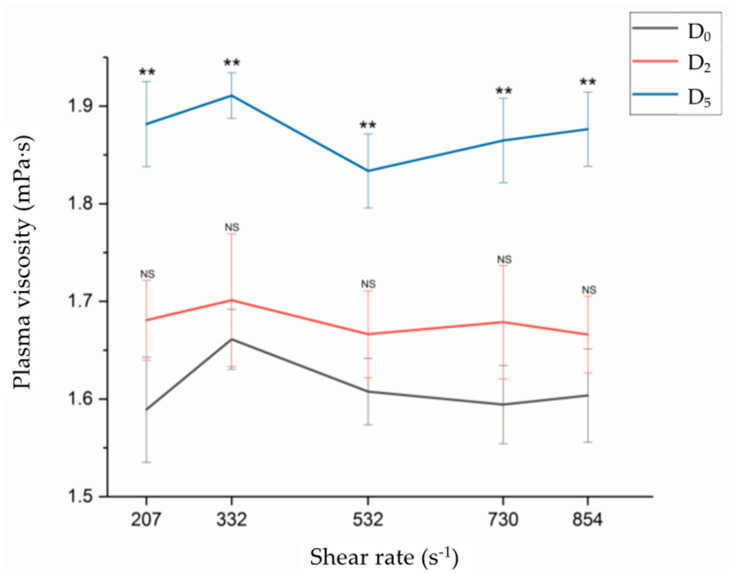
One-way ANOVA revealed no significant differences (NS) in plasma viscosity (PV) 2 days after surgery (D_2_) and before bypass (D_0_) at shear rates ranging from 207 to 854 s^−1^. However, there were significant differences in PV 5 days after surgery (D_5_) and just before surgery (D_0_) for each measurement shear rate (ANOVA with Tukey post hoc analysis). ** *p* < 0.01.

As depicted in Figure 1, the plasma viscosity (PV) demonstrated Newtonian fluid behavior within the shear rate range of 207–854 s^−1^. The mean PV values varied from 1.58 to 1.66 mPa·s before CABG surgery and from 1.66 to 1.70 mPa·s in the days after surgery, with no significant differences between the D_2_ and D_5_ time points. However, the mean PV values notably increased 5 days post-surgery (D_5_), ranging from 1.83 to 1.91 mPa·s and showing a significant elevation compared with both the D_0_ and D_2_ values (*p* < 0.01). This could explain the lack of a significant increase in PV at D_2_ compared with that at D_0_, despite the significant increase in fibrinogen (*p* = 0.015) from before CPB (D_0_) to day 2 (D_2_). As the hemodilution effect diminished on day 5 (D_5_), the PV became significantly higher than that at D_0_ (*p* < 0.001).

**Figure 2:**
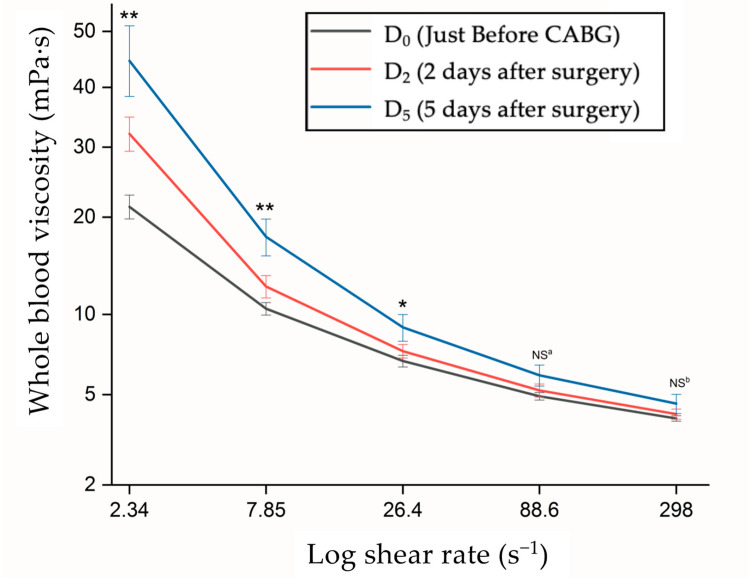
At shear rates of 2.34^−1^ and 7.85 s^−1^, we observed marked differences in whole blood viscosity (WBV), as confirmed by ANOVA (** *p* < 0.01). At 26.4 s^−1^, the difference between D_5_ and D_0_ was significant (ANOVA and Tukey-HSD post hoc analysis; * *p* < 0.05). However, no significant differences in mean WBV were found at 88.6 and 298 s^−1^ by ANOVA (NS ^a^, NS ^b^; both *p* > 0.05).

Figure 2 demonstrates that the whole blood viscosity (WBV) behaved as a non-Newtonian fluid. The WBV decreased with an increasing shear rate, but the change was minimal when the shear rate exceeded 50 s^−1^. Interestingly, the results revealed a special phenomenon in which both the PV and WBV reached their highest values at D_5_ compared with D_2_ and D_0_ within the shear rate range of 2.34–298 s^−1^. Analysis of variance (ANOVA) detected significant differences between the three phases (*p* < 0.01) at shear rates between 2.34 and 7.85 s^−1^. ANOVA also detected significant differences among the three measurements (*p* < 0.05) at a shear rate of 26.4 s^−1^ attributable to shear thinning behavior. Furthermore, the paired *t*-test confirmed significant differences between D_5_ and D_0_. However, no significant differences were identified at higher shear rates (88.6 s^−1^ and 298 s^−1^). 

These findings suggest notable alterations in the hemorheological properties of blood during the postoperative period, which may influence patient recovery and clinical management following CABG surgery. It is important to highlight that inflammation plays a pivotal role in driving these hemorheological changes, which may lead to enhanced plasma viscosity and WBV, potentially causing complications by altering blood flow dynamics.

### 4.3. Data of Blood Biochemistry

**Figure 3:**
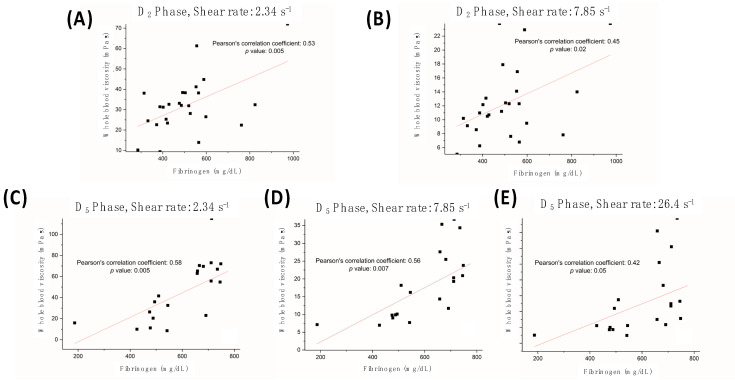
There are significant correlations between whole blood viscosity (WBV) and fibrinogen concentration at different shear rates, both on day 2 post-surgery (D_2_ (**A**,**B**); 2.34 s^−1^ and 7.85 s^−1^, respectively) and on day 5 post-surgery (D_5_ (**C**–**E**); 2.34, 7.85, and 26.4 s^−1^, respectively).

Blood biochemistry laboratory data, including inflammation-related parameters, are presented in Table 3. The mean hematocrit (%) decreased from 37.76 ± 5.13 at D_0_ to 30.63 ± 4.52 at D_2_ and remained at 29.98 ± 3.62 at D_5_ due to hemodilution during CPB. Although ANOVA revealed significant differences among the values of the three time points for various parameters, including those related to inflammation, only the fibrinogen levels demonstrated a significant correlation with WBV at D_2_ and D_5_ at shear rates below 26.4 s^−1^. Figure 3 displays the Pearson correlation coefficients for fibrinogen and WBV at different time points and shear rates, indicating a moderate-to-high correlation between these variables.

## 5. Discussion

Many cardiovascular events, such as coronary artery disease and stroke, have been intricately linked to blood rheological properties, but definitive causality has yet to be established [14,15]. Recent evidence suggests that plasma proteins may play a pivotal role in these relationships [16]. The existing literature on rheological changes in post-on-pump coronary artery bypass grafting (CABG) is limited [14,17]. The whole blood viscosity (WBV) is influenced by hematocrit, red blood cell aggregation, and deformability, as well as the plasma viscosity (PV), whereas the PV is determined by macromolecular compounds. Hematocrit has the most significant impact on the WBV.

Balancing the hematocrit during CPB is still a subject of debate. In our study, the balanced electrolyte solution used to prime the extracorporeal perfusion circuit resulted in a hematocrit of 25–30% [18,19]. In our series, the hemodilution caused by the 1.5–2.0 L of balanced electrolyte solution, usually lactated Ringers, was used to prime the extracorporeal perfusion circuit. Hemodilution led to decreased WBV during surgery for improved microcirculatory flow and red cell rigidity [20], but the WBV unexpectedly increased post-CABG, which could indicate the involvement of other factors such as inflammation and alterations in blood cell properties. Inflammation can alter blood rheology by affecting plasma proteins, activating blood cells, and modifying coagulation factors, all of which may contribute to the risk of postoperative complications [9,21]. Understanding the interplay between inflammation, hemorheological characteristics, and post-surgical complications could offer insights into potential therapeutic interventions, thereby enhancing clinical decision making and patient prognosis post-CABG. For instance, elevated inflammatory markers (i.e., fibrinogen, lipoproteins, immunoglobulins, C-reactive protein, activated platelets, white blood cells, and fibrinogen) may contribute to endothelial impairment, platelet activation, and a hypercoagulable state, thereby increasing the risk of thrombotic incidents or other postoperative complications. These inflammatory proteins could enhance erythrocyte aggregation and reduce its deformability, thereby increasing blood viscosity in postoperative thrombotic events such as adverse neurologic outcomes [22].

Otto et al. reported that fibrinogen was correlated with hemorheology, especially PV [23]. Contrary to these findings, our study did not find any significant correlation between fibrinogen and PV at any of the three time points measured. We propose that this lack of correlation may be attributed to other variables, such as lipoproteins and immunoglobulins, which were also diluted during CPB. As hemodilution diminished almost one week post-CABG (D_5_), the PV became significantly higher. This suggests that inflammation could modify the blood rheological properties post-surgery, potentially contributing to postoperative complication risks.

Regarding the relationship between fibrinogen and WBV following CPB (Figure 3), Ramunni et al. reported that erythrocyte aggregation and fibrinogen increased in parallel and concluded that a decrease in fibrinogen concentration would result in a corresponding decrease in blood viscosity [24]. Our findings also indicated a strong correlation between the WBV and fibrinogen after CABG at shear rates below 26.4 s^−1^ (Figure 2). The viscosity at this low shear rate region closely approximates the diastolic blood viscosity (DBV) [25,26], which is indicative of the low blood flow at the occluded vascular segment. The viscosity is largely influenced by the aggregation of red blood cells within small vessels [27]. This phenomenon can be attributed to the formation of red blood cell rouleaux at this low blood flow, with the blood viscosity being primarily determined by both the concentration and aggregation properties of these cells. Consequently, intensified aggregation of red blood cells could exacerbate flow disturbances and elevate the DBV, thereby triggering endothelial remodeling, luminal occlusion, and acute lacunar infarction [28].

Temperature fluctuations also impact viscosity. In the study, hypothermia was mild during on-pump CABG. Although the WBV was measured at a constant 37 °C, temperature variation before and after on-pump CABG might have affected the WBV by independently altering erythrocyte aggregation and deformability [29]. In a series of pediatric patients, significant changes in viscosity and elasticity occurred during normothermic and hypothermic cardiac surgery after 1 h, and viscoelasticity was also shown to slightly increase 24 h post cardiac surgery, consistent with the results of our study. Both the PV and WBV should be measured, along with changes in erythrocytes during hypothermia and rewarming to normothermia in future studies.

Understanding the relationship between inflammation and blood viscosity could offer therapeutic targets and strategies to reduce post-CABG complications. In this longitudinal study, we noted that the effects of inflammation on blood’s physical properties are not static but evolve over the course of the peri-operative and post-operative periods. However, this study has some limitations that should be considered. The viscosity fluctuates in vivo in different types of blood vessels. In this work, the viscosity was measured in vitro, which may not fully reflect the actual viscosity under physiological conditions. Furthermore, given the small sample size of this study, it is crucial to acknowledge that a power analysis was conducted to determine the sample size’s appropriateness. Nevertheless, this study’s primary aim was to identify the preliminary trends and associations between the variables under investigation, serving as a foundation for more extensive, more definitive future studies. Despite these limitations, this study provides valuable insights into the changes in blood rheology following CABG and highlights the potential role of inflammation in modulating these changes. For example, the use of anti-inflammatory drugs such as corticosteroids, non-steroidal anti-inflammatory drugs (NSAIDs) [30,31,32,33], pentoxifylline (Trental), or *Ginkgo biloba* [34] could be explored post surgery to mitigate the hypercoagulable state often observed in CABG patients. Additionally, anticoagulant therapies like low molecular weight heparin could be considered in conjunction with anti-inflammatory treatments to provide a multi-faceted approach to reducing postoperative complications [35]. Thus, this study raises the possibility of personalized medicine approaches, where treatments could be tailored based on an individual patient’s inflammatory markers and blood rheological properties. Further research is needed to understand the underlying pathogenetic mechanisms better and develop targeted interventions to reduce the risk of complications associated with altered blood rheology after CABG.

## 6. Conclusions

In patients undergoing on-pump CABG, we observed modest elevations in both PV and WBV, with the PV rising until approximately one week post surgery and the WBV persisting for a similar duration. Notably, a significant correlation was found between fibrinogen, an acute-phase reactant, and the WBV at shear rates below 26.4 s^−1^, indicating the possible involvement of inflammation in influencing hemorheological properties during the postoperative period. Inflammation could potentially affect the blood viscosity by modulating plasma proteins, activating blood cells, and adjusting coagulation factors.

Understanding the complex hemodynamics between inflammation and hemorheological properties holds promise for refining patient care and may potentially reveal therapeutic targets to enhance outcomes post CABG surgery. Future research is necessary to delve deeper into the possible connections between inflammation, blood rheological properties, and postoperative complications and better understand CPB’s effects on erythrocyte aggregation and deformability, ultimately aiming for enhanced patient outcomes after CABG.

## Figures and Tables

**Table 1:** Patient demographic and clinical characteristics.

Characteristics	All Patients (N = 25) Case (%)
Male/Female	17/8
Age (years)	63.1 ± 3.1
BMI (kg/m^2^)	24.16 ± 0.81
Smoking	16 (64%)
Alcohol consumption	3 (12%)
LVEF (%)	57.6 ± 2.96
Hypertension	16 (64%)
Hyperlipidemia	15 (60%)
Diabetes	9 (36%)
Stroke	4 (16%)
COPD	1 (4%)
Aspirin administration	20 (80%)
Clopidogrel administration	4 (16%)

BMI = body mass index; COPD = chronic obstructive pulmonary disease; LVEF = left ventricular ejection fraction; smoking and alcohol consumption = current tobacco and alcohol consumption status; hypertension = medication for hypertension or a blood pressure ≥140/90 mmHg on repeated measurement; hyperlipidemia = on lipid-lowering medications or an overnight fasting cholesterol level ≥220 mg/dL or low-density lipoprotein cholesterol level ≥140 mg/dL; diabetes mellitus = medication for diabetes or a fasting blood sugar ≥126 mg/dL.

**Table 2:** Peri-operative variables and post-operative outcomes.

Parameters	All Patients (N = 25) Number (%)
Peri-operative variables	
Graft number	3.81 ± 0.23
LIMA harvesting	23 (92%)
Cross-clamping time (min)	55.4 ± 13.1
Pump time (min)	124.0 ± 8.3
Procedure time (min)	344.8 ± 17.2
Packed red blood cells (PRBC, units)	3.76 ± 0.60
Fresh frozen plasma (FFP, units)	4.44 ± 1.00
Platelet concentrate (units)	1.44 ± 0.58
Post-operative outcomes	
24 h drainage amount (mL)	236.7 ± 48.9
Packed red blood cells (PRBC, units)	0.84 ± 0.37
Fresh frozen plasma (FFP, units)	1.12 ± 0.44
Platelet concentrate (units)	0.08 ± 0.28
Ventilation period (days)	1.40 ± 0.32
ICU stay (days)	2.75 ± 0.98
Hospital stay (days)	10.90 ± 4.18
Lung atelectasis	1 (4%)
Acute kidney injury	2 (8%)

LIMA = left internal mammary artery; ICU = intensive care unit; acute kidney injury = increase in serum creatinine of more than or equal to 0.3 mg/dL.

**Table 3:** One-way ANOVA with Tukey’s post hoc or paired *t*-test for laboratory data before CABG surgery (D_0_), 2 days after surgery (D_2_), and 5 days after surgery (D_5_).

Parameters	D_0_	D_2_	D_5_	*p* Value	Post Hoc *Tukey-HSD* *Test with a* *Significant Difference*
White blood cell (10^3^/uL)	6.63 ± 2.23	9.61 ± 3.17	8.48 ± 2.45	<0.001	D_5_ versus D_0_, D_2_ versus D_0_
Platelet (10^3^/uL)	189.4 ± 44.7	129.8 ± 25.6	267.2 ± 94.0	<0.001	D_5_ versus D_0_,
Hemoglobin (g/dL)	12.71 ± 1.90	10.48 ± 1.36	9.89 ± 1.08	<0.001	D_5_ versus D_0_,
Hematocrit (%)	37.76 ± 5.13	30.63 ± 4.52	29.98 ± 3.62	<0.001	--
Prothrombin time (s)	10.63 ± 1.40	10.94 ± 0.73	11.34 ± 2.16	0.26	--
Partial thromboplastin time (PTT) (s)	29.60 ± 2.87	28.89 ± 3.02	31.37 ± 3.84	0.02	--
International normalized ratio	0.97 ± 0.24	1.05 ± 0.09	1.07 ± 0.24	0.20	--
Creatine kinase (U/L)	86.5 ± 44.5	756.8 ± 383.9	NA	<0.001	--
Troponin-I (ng/mL)	0.77 ± 1.11	6.23 ± 4.84	NA	<0.001	--
Aspartate aminotransferase (U/L)	26.7 ± 14.2	55.8 ± 20.9	NA	<0.001	--
Alanine transaminase (U/L)	22.8 ± 11.4	28.0 ± 16.2	NA	0.20	--
Total bilirubin (mg/dL)	0.94 ± 0.50	1.08 ± 0.39	NA	0.25	--
Blood urea nitrogen (mg/dL)	20.8 ± 7.5	21.0 ± 9.0	20.8 ± 12.9	0.99	--
Creatinine(mg/dL)	1.12 ± 0.92	1.12 ± 0.57	1.15 ± 0.96	0.99	--
Blood urea nitrogen/Creatinine ratio	20.5 ± 5.4	19.3 ± 5.1	18.9 ± 3.3	0.48	--
Sodium (mmol/L)	136.5 ± 3.4	142.9 ± 2.9	137.4 ± 3.8	<0.001	D_2_ versus D_0_
Potassium (mmol/L)	3.73 ± 0.35	3.70 ± 0.31	3.75 ± 0.43	0.88	--
C-reactive protein (mg/dL)	0.70 ± 1.51	7.42 ± 8.00	5.76 ± 6.30	<0.001	D_5_ versus D_0_, D_2_ versus D_0_
Fibrinogen (mg/dL)	411.3 ± 108.4	509.3 ± 159.2	601.7 ± 145.7	<0.001	D_5_ versus D_0_, D_2_ versus D_0_

NA = not available.

## Data Availability

Not applicable.

## Abbreviations

CABG = coronary artery bypass grafting; LVEF = left ventricular ejection fraction; PV = plasma viscosity; WBV = whole blood viscosity; CPB = cardiopulmonary bypass.
